# Supplementary material for: SIRT2i_Predictor: A Machine Learning-Based Tool to Facilitate the Discovery of Novel SIRT2 Inhibitors
Source: Pharmaceuticals (Basel). 2023 Jan 14;16(1):127. doi: 10.3390/ph16010127 (PMC9864763; doi:10.3390/ph16010127)
Supplement: Supplementary file 1 [file pharmaceuticals-16-00127-s001.zip › pharmaceuticals-2127403-supplementary.pdf]

SUPPORTING INFORMATION

SIRT2i_Predictor: A Machine Learning-Based Tool to Facilitate the Discovery of Novel SIRT2 Inhibitors

Nemanja Djokovic [1,*], Minna Rahnasto-Rilla [2], Nikolaos Lougiakis [3], Maija Lahtela-Kakkonen [2] and Katarina Nikolic [1,*]

1. Department of Pharmaceutical Chemistry, Faculty of Pharmacy, University of Belgrade, Vojvode Stepe 450, 11221 Belgrade, Serbia
2. School of Pharmacy, University of Eastern Finland, P.O. Box 1627, 70210 Kuopio, Finland
3. Laboratory of Medicinal Chemistry, Section of Pharmaceutical Chemistry, Department of Pharmacy, School of Health Sciences, National and Kapodistrian University of Athens, Panepistimiopolis-Zografou, 15771 Athens, Greece

* Correspondence: nemanja.djokovic@pharmacy.bg.ac.rs (N.D.); katarina.nikolic@pharmacy.bg.ac.rs (K.N.)

Table of Contents

Table S1 1
Table S2. 3
Table S4. 7
Table S5. 9
Table S6 10
Table S7. 12
Table S8. 13
Table S9. 14
Table S10. 15
Table S11. 16
Table S12. 17
Supplementary Note S1 – Internal validation parameters 18
Supplementary Note S2 - Supplementary methods 18

Table S1. List of hyperparameters, their values and objectives considered for optimization through Bayesian search.

Model	ML algorithm	Tuned hyperparameters
Regression	RF	{'bootstrap': [True, False],'max_depth': [5, 20, 35, 50, 65, 80, None], 'max_features': ['log2', 'sqrt'], 'min_samples_leaf': [2, 4, 6], 'min_samples_split': [2, 5, 10], 'n_estimators': [250,500,750,1000,1250,1500]}, scoring : (' r2')
	SVR	{'C': [0.01, 0.1, 1, 10, 100, 200, 400, 1000], 'gamma': [100,10,1,1e-1,1e-2, 1e-3], 'kernel': ['rbf', 'linear']}, scoring : (' r2')
	XGBoost	{'learning_rate': [0.01, 0.1], 'max_depth': [3, 6, 10], 'min_child_weight': [1, 3, 5], 'gamma': [0, 1, 5], 'subsample': [0.6, 0.7, 0.8, 0.9, 1.0], 'colsample_bytree': [0.5, 0.6, 0.7, 0.8, 0.9, 1.0], 'n_estimators': [500, 1500]}, scoring : (' r2')
	KNN	{'n_neighbors': [5,9,15,19,25,35,45,55,71], 'weights': ['distance'],'p': [1,2],'metric': ['jaccard', 'dice', 'rogerstanimoto']}, scoring : ('r2')
	DNN	{number of Dense layers: (1-8, activation = 'relu'; None in output layer), number of neurons in each layer: ((100 - 5000), step = (50 - 1000)), added dropouts: ((0 -0.5), step = 0.1), SGD optimizer learning rate: (1e-4 to 1e-2, sampling = ('log', default = 1e-3)), loss_fn : 'MeanSquaredError', batch_size : (32-128, step = 32, default = 64), objective : ('val_mean_squared_error','min'), used 30 % of training set as validation set }
Binary	RF	{'bootstrap': [True, False], 'max_depth': [5, 20, 35, 50, 65, 80, None], 'max_features': ['log2', 'sqrt'], 'min_samples_leaf': [2, 4, 6], 'min_samples_split': [2, 5, 10], 'n_estimators': [250,500,750,1000,1250,1500]}, scoring : (' balanced_accuracy')
	SVC	{'C': [0.01, 0.1, 1, 10, 100, 200, 400, 1000], 'gamma': [100,10,1,1e-1,1e-2, 1e-3], 'kernel': ['linear','rbf']}, scoring : (' balanced_accuracy')
	XGBoost	{'learning_rate': [0.01, 0.1], 'max_depth': [3, 6, 10], 'min_child_weight': [1, 3, 5], 'gamma': [0, 1, 5], 'subsample': [0.6, 0.7, 0.8, 0.9, 1.0], 'colsample_bytree': [0.5, 0.6, 0.7, 0.8, 0.9, 1.0], 'n_estimators': [500, 1500]}, scoring : (' balanced_accuracy')
	KNN	{'n_neighbors': [5,9,15,19,25,35,45,55,71], 'weights': ['distance'],'p': [1,2],'metric': ['jaccard', 'dice', 'rogerstanimoto']}, scoring : (' balanced_accuracy')
	DNN	{number of Dense layers: (1-8, (activation = 'relu'; 'sigmoid' in output layer)), number of neurons in each layer: ((100 - 5000), step = (50 - 1000)), added dropouts: ((0-0.5), step = 0.1), Adam optimizer learning rate: ((1e-4 to 1e-2), sampling = 'log', default = 1e-3), loss_fn : 'BinaryCrossentropy', batch_size : (32-128, step = 32, default = 64), objective : ('val_binary_accuracy','max'), used 30 % of training set as validation set }
Multiclass	RF	{'bootstrap': [True, False], 'max_depth': [5, 20, 35, 50, 65, 80, None], 'max_features': ['log2', 'sqrt'], 'min_samples_leaf': [2, 4, 6], 'min_samples_split': [2, 5, 10], 'n_estimators': [250,500,750,1000,1250,1500]}, scoring : (' balanced_accuracy')

	**SVC**	{'C': [0.01, 0.1, 1, 10, 100, 200, 400, 1000], 'gamma': [100,10,1,1e-1,1e-2, 1e-3], 'kernel': ['linear', 'rbf']}, scoring : (' balanced_accuracy')
	**XGBoost**	{'learning_rate': [0.01, 0.1], 'max_depth': [3, 6, 10], 'min_child_weight': [1, 3, 5], 'gamma': [0, 1, 5], 'subsample': [0.6, 0.7, 0.8, 0.9, 1.0], 'colsample_bytree': [0.5, 0.6, 0.7, 0.8, 0.9, 1.0], 'n_estimators': [500, 1500]}, scoring : (' balanced_accuracy')
	**KNN**	{'n_neighbors': [5,9,15,19,25,35,45,55,71], 'weights': ['distance'],'p': [1,2],'metric': ['jaccard', 'dice', 'rogerstanimoto']}, scoring : (' balanced_accuracy')
	**DNN**	{number of Dense layers: ((1-8), activation = ('relu'; 'softmax' in outplut layer)), number of neurons in each layer: ((100 - 5000), step = (50 - 1000)), added dropouts: (0 -0.5, step = 0.1), Adam optimizer learning rate: (1e-4 to 1e-2, sampling = ('log', default = 1e-3)), loss_fn : 'SparseCategoricalCrossentropy', batch_size : ((32-128), step = 32, default = 64), objective : ('val_categorical_accuracy','max'), used 30 % of training set as validation set}

Table S2. Optimized hyperparameters for final models included in SIRT2i_Predictor.

Final model	Optimized hyperparameters
Regression XGBoost:ECFP4	[('colsample_bytree', 0.5), ('gamma', 0), ('learning_rate', 0.01), ('max_depth', 10), ('min_child_weight', 3), ('n_estimators', 1500), ('subsample', 0.6)]
Binary RF:ECFP4	[('bootstrap', False), ('max_depth', 65), ('max_features', 'sqrt'), ('min_samples_leaf', 2), ('min_samples_split', 5), ('n_estimators', 1000)]
Multiclass SIRT1/2 RF:ECFP4	[('bootstrap', False), ('max_depth', 80), ('max_features', 'sqrt'), ('min_samples_leaf', 2), ('min_samples_split', 5), ('n_estimators', 750)]
Multiclass SIRT2/3 DNN:descriptors model	InputLayer : 'config': {'batch_input_shape': (None, 316), 'units': 4000, 'activation': 'relu'}<br>Dense Layer 1 : 'config': {'name': 'dense_1', 'units': 100, 'activation': 'relu'}<br>Dropout Layer : 'config': {'name': 'dropout', 'rate': 0.0}<br>Dense Layer 2 : 'config': {'name': 'dense_2', 'units': 3, 'activation': 'softmax'}<br>Optimizer: {'name': 'Adam', 'learning_rate': 1e-04, 'decay': 0.0, 'beta_1': 0.9, 'beta_2': 0.999, 'epsilon': 1e-07, 'amsgrad': False}<br>batch_size = 32<br>Loss function: SparseCategoricalCrossentropy

Table S3. Mordred descriptors selected after feature selection procedure.

Model Type	List of selected Mordred descriptors
**Regression models**	nAromAtom, ATS0Z, AATS5d, AATS6v, AATS4i, AATS5i, ATSC7dv, ATSC6d, ATSC8d, ATSC7Z, ATSC1v, ATSC2v, ATSC1se, ATSC2i, ATSC4i, ATSC8i, AATSC2dv, AATSC5dv, AATSC0d, AATSC3se, AATSC5se, MATS1p, MATS6p, GATS4dv, GATS6d, GATS4Z, GATS3v, GATS5v, GATS6se, GATS4i, GATS5i, C1SP2, Xc-5d, AXp-1d, NaaS, SaasC, IC2, IC3, PEOE_VSA3, PEOE_VSA8, PEOE_VSA12, SMR_VSA6, SlogP_VSA10, EState_VSA2, VSA_EState1, n10FRing, SLogP, JGI2, JGI6, JGI8, JGI9, TopoShapeIndex
**Binary classification models**	AATS0dv, AATS1dv, AATS0d, AATS5d, AATS6d, AATS0Z, AATS1Z, AATS5Z, AATS6Z, AATS0v, AATS5v, AATS6v, AATS0se, AATS5se, AATS6se, AATS0i, AATS3i, AATS4i, AATS5i, AATS6i, ATSC1dv, ATSC2dv, ATSC3dv, ATSC4dv, ATSC5dv, ATSC6dv, ATSC7dv, ATSC8dv, ATSC1d, ATSC2d, ATSC3d, ATSC4d, ATSC5d, ATSC6d, ATSC7d, ATSC8d, ATSC1Z, ATSC2Z, ATSC3Z, ATSC4Z, ATSC5Z, ATSC6Z, ATSC7Z, ATSC8Z, ATSC1v, ATSC2v, ATSC3v, ATSC4v, ATSC5v, ATSC6v, ATSC7v, ATSC8v, ATSC0se, ATSC1se, ATSC2se, ATSC3se, ATSC4se, ATSC5se, ATSC6se, ATSC7se, ATSC8se, ATSC1i, ATSC2i, ATSC3i, ATSC4i, ATSC5i, ATSC6i, ATSC7i, ATSC8i, AATSC1dv, AATSC2dv, AATSC4dv, AATSC5dv, AATSC6dv, AATSC0d,

	AATSC1d, AATSC2d, AATSC5d, AATSC6d, AATSC5Z, AATSC6Z, AATSC0v, AATSC1v, AATSC6v, AATSC0se, AATSC2se, AATSC3se, AATSC4se, AATSC6se, AATSC1pe, AATSC0i, MATS5Z, MATS1p, MATS4p, MATS6p, GATS1dv, GATS2dv, GATS3dv, GATS5dv, GATS1d, GATS2d, GATS4d, GATS5d, GATS6d, GATS5Z, GATS6Z, GATS2v, GATS6v, GATS1se, GATS2se, GATS4se, GATS5se, GATS6se, GATS1p, GATS2p, GATS3p, GATS6i, BalabanJ, Xc-6dv, Xpc-4d, Xpc-4dv, AXp-1d, AXp-0dv, AXp-1dv, NsCH3, NdCH2, NtCH, NdsCH, NaaCH, NsssCH, NdssC, NaasC, NaaaC, NssssC, NsNH2, NdNH, NssNH, NaaNH, NdsN, NaaN, NsssN, NddsN, NaasN, NsOH, NssO, NaaO, NsSH, NdS, NssS, NaaS, NddssS, NdSe, SssCH2, SsssCH, SdssC, SaasC, SssssC, ECIndex, fragCpx, fMF, nHBAcc, nHBDon, IC0, IC1, IC2, IC3, SIC0, SIC1, SIC2, SIC3, MIC0, MIC1, ZMIC1, Lipinski, GhoseFilter, FilterItLogS, PEOE_VSA2, PEOE_VSA3, PEOE_VSA4, PEOE_VSA5, PEOE_VSA6, PEOE_VSA7, PEOE_VSA8, PEOE_VSA9, PEOE_VSA10, PEOE_VSA11, PEOE_VSA12, PEOE_VSA13, SMR_VSA2, SMR_VSA3, SMR_VSA4, SMR_VSA6, SMR_VSA9, SlogP_VSA3, SlogP_VSA4, SlogP_VSA8, SlogP_VSA10, EState_VSA2, EState_VSA3, EState_VSA4, EState_VSA5, EState_VSA6, EState_VSA7, EState_VSA8, EState_VSA9, VSA_EState1, VSA_EState2, VSA_EState3, VSA_EState5, VSA_EState8, VSA_EState9, MDEC-23, MDEC-33, nRing, n7Ring, nG12Ring, nFRing, n8FRing, n9FRing, nFHRing, nG12FaRing, nFaHRing, n9FARing, n10FARing, SLogP, JGI1, JGI2, JGI3, JGI4, JGI5, JGI6, JGI7, JGI8
**Multiclass Classification Models SIRT1/2**	nAromAtom, nSpiro, nBridgehead, nHetero, nN, nO, nS, nP, nF, nCl, nBr, nI, nX, ATS0Z, AATS0dv, AATS0d, AATS5d, AATS6d, AATS0Z, AATS1Z, AATS5Z, AATS6Z, AATS4v, AATS5v, AATS6v, AATS0se, AATS5se, AATS6se, AATS0p, AATS0i, AATS3i, AATS4i, AATS5i, AATS6i, ATSC1dv, ATSC2dv, ATSC3dv, ATSC4dv, ATSC5dv, ATSC6dv, ATSC7dv, ATSC8dv, ATSC1d, ATSC2d, ATSC3d, ATSC4d, ATSC5d, ATSC6d, ATSC7d, ATSC8d, ATSC1Z, ATSC2Z, ATSC3Z, ATSC4Z, ATSC5Z, ATSC6Z, ATSC7Z, ATSC8Z, ATSC1v, ATSC2v, ATSC3v, ATSC4v, ATSC5v, ATSC6v, ATSC7v, ATSC8v, ATSC1se, ATSC2se, ATSC3se, ATSC4se, ATSC5se, ATSC6se, ATSC7se, ATSC8se, ATSC1p, ATSC3p, ATSC4p, ATSC5p, ATSC6p, ATSC7p, ATSC8p, ATSC1i, ATSC2i, ATSC3i, ATSC4i, ATSC5i, ATSC6i, ATSC7i, ATSC8i, AATSC0dv, AATSC1dv, AATSC2dv, AATSC3dv, AATSC4dv, AATSC5dv, AATSC6dv, AATSC0d, AATSC1d, AATSC2d, AATSC5d, AATSC6d, AATSC5Z, AATSC6Z, AATSC0v, AATSC1v, AATSC2v, AATSC6v, AATSC1se, AATSC2se, AATSC3se, AATSC4se, AATSC5se, AATSC6se, AATSC0p, AATSC2p, AATSC6p, AATSC2i, MATS4Z, GATS1dv, GATS4dv, GATS3d, GATS4Z, GATS5Z, GATS2v, GATS6v, GATS1se, GATS2se, GATS3se, GATS4se, GATS5se, GATS1p, GATS2p, GATS4p, GATS5p, GATS6i, Xch-6d, Xch-5dv, Xc-3d, Xc-4d, Xc-5d, Xc-6d, Xc-3dv, Xc-4dv, Xc-5dv, Xc-6dv, Xpc-4d, Xpc-4dv, AXp-1d, AXp-0dv, AXp-1dv, NsCH3, NdCH2, NtCH, NdsCH, NaaCH, NsssCH, NaasC, NaaaC, NssssC, NsNH2, NdNH, NssNH, NaaNH, NdsN, NaaN, NsssN, NddsN, NaasN, NsOH, NssO, NaaO, NsSH, NdS, NssS, NaaS, NddssS, NdSe, SssCH2, SdssC, SaasC, SssssC, ECIndex, fragCpx, fMF, nHBDon, IC0, IC1, IC2, IC3, SIC0, SIC1, SIC2, SIC3, MIC0, MIC1, Lipinski, GhoseFilter, FilterItLogS, PEOE_VSA2, PEOE_VSA3, PEOE_VSA4, PEOE_VSA5,

	PEOE_VSA6, PEOE_VSA7, PEOE_VSA8, PEOE_VSA9, PEOE_VSA10, PEOE_VSA11, PEOE_VSA12, PEOE_VSA13, SMR_VSA2, SMR_VSA3, SMR_VSA4, SMR_VSA6, SMR_VSA9, SlogP_VSA3, SlogP_VSA4, SlogP_VSA8, SlogP_VSA10, SlogP_VSA11, EState_VSA2, EState_VSA3, EState_VSA4, EState_VSA6, EState_VSA7, EState_VSA8, EState_VSA9, VSA_EState1, VSA_EState3, VSA_EState5, VSA_EState7, VSA_EState8, VSA_EState9, MDEC-23, MDEC-33, piPC3, nRing, n7Ring, n8Ring, nG12Ring, nHRing, naHRing, n6aHRing, nARing, nAHRing, n6AHRing, nFRing, n9FRing, nG12FRing, nFHRing, n10FHRing, nFaRing, n9FaRing, n10FaRing, nG12FaRing, nFARing, n9FARing, n10FARing, nFAHRing, n9FAHRing, n10FAHRing, RotRatio, SLogP, JGI1, JGI2, JGI3, JGI4, JGI5, JGI6, JGI7
**Multiclass Classification Models SIRT2/3**	AATS0dv, AATS5d, AATS6d, AATS0Z, AATS1Z, AATS6Z, AATS5v, AATS6v, AATS0se, AATS5se, AATS6se, AATS0p, AATS4p, AATS5p, AATS6p, AATS0i, AATS1i, AATS3i, AATS4i, AATS5i, AATS6i, ATSC2c, ATSC3c, ATSC5c, ATSC6c, ATSC7c, ATSC8c, ATSC1dv, ATSC2dv, ATSC3dv, ATSC4dv, ATSC5dv, ATSC6dv, ATSC7dv, ATSC8dv, ATSC1d, ATSC2d, ATSC3d, ATSC4d, ATSC5d, ATSC6d, ATSC7d, ATSC8d, ATSC1Z, ATSC2Z, ATSC3Z, ATSC4Z, ATSC5Z, ATSC6Z, ATSC7Z, ATSC8Z, ATSC1v, ATSC2v, ATSC3v, ATSC4v, ATSC5v, ATSC6v, ATSC7v, ATSC8v, ATSC1se, ATSC2se, ATSC3se, ATSC4se, ATSC5se, ATSC6se, ATSC7se, ATSC8se, ATSC1pe, ATSC1p, ATSC3p, ATSC4p, ATSC5p, ATSC6p, ATSC7p, ATSC8p, ATSC1i, ATSC2i, ATSC3i, ATSC4i, ATSC5i, ATSC6i, ATSC7i, ATSC8i, AATSC0c, AATSC1c, AATSC2c, AATSC3c, AATSC4c, AATSC5c, AATSC6c, AATSC1dv, AATSC2dv, AATSC3dv, AATSC4dv, AATSC5dv, AATSC6dv, AATSC0d, AATSC1d, AATSC2d, AATSC4d, AATSC5d, AATSC6d, AATSC3Z, AATSC5Z, AATSC6Z, AATSC0v, AATSC1v, AATSC2v, AATSC6v, AATSC1se, AATSC2se, AATSC3se, AATSC4se, AATSC5se, AATSC6se, AATSC1pe, AATSC0p, AATSC1p, AATSC6p, AATSC2i, AATSC6i, MATS1c, MATS3Z, MATS4Z, MATS5Z, MATS6Z, MATS2se, GATS2c, GATS3c, GATS4c, GATS5c, GATS6c, GATS1dv, GATS2dv, GATS3dv, GATS4dv, GATS5dv, GATS6dv, GATS1d, GATS2d, GATS3d, GATS4d, GATS5d, GATS6d, GATS1Z, GATS2Z, GATS3Z, GATS4Z, GATS5Z, GATS6Z, GATS2v, GATS6v, GATS1se, GATS2se, GATS3se, GATS4se, GATS5se, GATS6se, GATS1p, GATS2p, GATS4p, GATS6p, GATS1i, GATS3i, GATS4i, GATS6i, BalabanJ, nBondsD, nBondsT, nBondsKD, RNCG, RPCG, C1SP1, C2SP1, C1SP2, C2SP2, C3SP2, C1SP3, C3SP3, C4SP3, Xch-3d, Xch-4d, Xch-5d, Xch-6d, Xch-6dv, Xc-3d, Xc-4d, Xc-5d, Xc-6d, Xc-3dv, Xc-4dv, Xc-5dv, Xc-6dv, AXp-1d, AXp-0dv, NsCH3, NtCH, NdsCH, NaaCH, NaasC, NaaaC, NssssC, NsNH2, NdNH, NaaNH, NdsN, NaaN, NsssN, NddsN, NaasN, NsOH, NssO, NaaO, NsSH, NdS, NssS, NaaS, NddssS, SssCH2, SaasC, SssssC, ECIndex, fragCpx, fMF, IC0, IC1, IC2, IC3, SIC0, SIC1, SIC3, MIC0, MIC1, Lipinski, GhoseFilter, FilterItLogS, PEOE_VSA2, PEOE_VSA3, PEOE_VSA4, PEOE_VSA5, PEOE_VSA6, PEOE_VSA7, PEOE_VSA8, PEOE_VSA9, PEOE_VSA10, PEOE_VSA11, PEOE_VSA13, SMR_VSA2, SMR_VSA4, SMR_VSA6, SMR_VSA9, SlogP_VSA3, SlogP_VSA4, SlogP_VSA8, SlogP_VSA10, SlogP_VSA11, EState_VSA2, EState_VSA3, EState_VSA4, EState_VSA5, EState_VSA6, EState_VSA7, EState_VSA8, EState_VSA9,

	VSA_EState1, VSA_EState3, VSA_EState7, VSA_EState8, MDEC-22, MDEC-23, MDEC-33, piPC3, nRing, n4Ring, n6Ring, n7Ring, nG12Ring, nHRing, n6HRing, naHRing, n6aHRing, nARing, n5ARing, n6ARing, nAHRing, n5AHRing, n6AHRing, nFRing, n8FRing, n9FRing, n10FRing, nG12FRing, nFHRing, n10FHRing, n9FaRing, n10FaRing, n10FaHRing, nFARing, n9FARing, n10FARing, nFAHRing, n9FAHRing, n10FAHRing, RotRatio, SLogP, JGI1, JGI2, JGI3, JGI4, JGI5, JGI6, JGI7, JGI8, JGI9, JGI10, TopoShapeIndex

Table S4. Results of internal (train set (int) and cross validation (CV)) and external (ext) validation for regression models.

ML algorithm	Molecular feature	R^2_{int}	$RMSE_{int}$	Q^2	$RMSE_{CV}$	R^2_{ext}	$RMSE_{ext}$	r^2_m	$\bar{r}^2_m$	Δr^2_m	Q^2_{F1}	Q^2_{F2}	Q^2_{F3}	CCC	Criteria 1[a]	Criteria 2[b]	Criteria 3[c]	k (or k')[d]
RF	descriptors	0.99	0.11	0.7 +/- 0.02	0.55 +/- 0.01	0.7	0.55	0.7	0.52	0.27	0.7	0.7	0.7	0.81	0.008	0.300	0.206	1.000 (0.990)
	ECFP4	0.9	0.31	0.74 +/- 0.03	0.5 +/- 0.03	0.75	0.5	0.75	0.6	0.23	0.75	0.75	0.75	0.85	0.004	0.170	0.125	1.001 (0.991)
	MACCS	0.86	0.37	0.68 +/- 0.03	0.56 +/- 0.03	0.71	0.53	0.71	0.55	0.26	0.71	0.71	0.71	0.82	0.005	0.243	0.171	1.001 (0.991)
	ECFP6	0.93	0.26	0.75 +/- 0.03	0.49 +/- 0.03	0.77	0.48	0.77	0.62	0.21	0.77	0.77	0.76	0.86	0.003	0.139	0.104	1.001 (0.992)
SVM	descriptors	0.99	0.11	0.63 +/- 0.03	0.6 +/- 0.03	0.62	0.61	0.63	0.44	0.31	0.62	0.62	0.62	0.77	0.004	0.074	0.052	1.003 (0.989)
	ECFP4	0.98	0.15	0.74 +/- 0.03	0.51 +/- 0.03	0.74	0.51	0.74	0.63	0.13	0.74	0.74	0.73	0.84	0.004	0.476	0.296	0.997 (0.992)
	MACCS	0.96	0.21	0.66 +/- 0.05	0.57 +/- 0.04	0.68	0.57	0.68	0.55	0.21	0.68	0.68	0.68	0.81	0.001	0.173	0.116	1.002 (0.988)
	ECFP6	0.99	0.11	0.75 +/- 0.03	0.5 +/- 0.03	0.74	0.51	0.74	0.63	0.18	0.74	0.74	0.74	0.86	0.001	0.098	0.072	0.997 (0.995)
XGBoost	descriptors	0.99	0.09	0.7 +/- 0.02	0.55 +/- 0.01	0.67	0.58	0.66	0.53	0.25	0.68	0.68	0.68	0.82	0.001	0.089	0.065	0.999 (0.993)
	ECFP4*	0.97	0.17	0.74 +/- 0.02	0.5 +/- 0.02	0.79	046	0.79	0.7	0.17	0.74	0.74	0.74	0.86	9.06E-05	0.064	0.050	1.005 (0.998)
	MACCS	0.87	0.35	0.69 +/- 0.04	0.55 +/- 0.03	0.71	0.53	0.71	0.58	0.24	0.7	0.7	0.7	0.82	0.000	0.176	0.124	1.001 (0.990)
	ECFP6	0.99	0.1	0.73 +/- 0.02	0.52 +/- 0.02	0.73	0.52	0.73	0.62	0.2	0.73	0.73	0.73	0.87	0.000	0.115	0.084	0.993 (0.999)
KNN	descriptors	1	0.05	0.65 +/- 0.06	0.59 +/- 0.06	0.68	0.56	0.68	0.56	0.23	0.68	0.68	0.68	0.86	0.004	0.068	0.048	0.995 (0.997)
	ECFP4	1	0.07	0.7 +/- 0.04	0.54 +/- 0.04	0.74	0.51	0.75	0.64	0.13	0.74	0.74	0.74	0.87	0.000	0.180	0.123	0.986 (1.004)
	MACCS	0.97	0.17	0.6 +/- 0.07	0.63 +/- 0.05	0.6	0.63	0.6	0.47	0.16	0.6	0.6	0.6	0.79	0.014	0.216	0.123	0.991 (0.996)
	ECFP6	1	0.06	0.72 +/- 0.04	0.53 +/- 0.04	0.76	0.49	0.76	0.66	0.12	0.76	0.76	0.76	0.87	0.003	0.057	0.041	0.999 (0.993)
DNN	descriptors	0.98	0.16	0.79 +/- 0.07	0.45 +/- 0.06	0.66	0.58	0.68	0.57	0.03	0.66	0.66	0.66	0.81	0.032	0.053	0.015	1.005 (0.985)
	ECFP4	0.99	0.09	0.85 +/- 0.07	0.37 +/- 0.07	0.74	0.51	0.74	0.63	0.18	0.73	0.73	0.73	0.84	0.000	0.108	0.079	1.002 (0.989)
	MACCS	0.93	0.27	0.64+/-0.07	0.59+/-0.05	0.68	0.56	0.69	0.56	0.16	0.68	0.68	0.67	0.80	0.007	0.123	0.079	1.004 (0.985)
	ECFP6	0.96	0.19	0.82 +/- 0.05	0.41 +/- 0.07	0.73	0.52	0.74	0.63	0.17	0.73	0.73	0.73	0.81	0.048	0.571	0.380	1.170 (0.832)

* Final model statistics
[a] $(R^2 - R_0^2)/ R^2 < 0.1$
[b] $(R^2 - R'^2_0)/ R^2 < 0.1$
[c] $\left| R^2 - R'^2_0 \right| < 0.3$
[d] $0.85 \le k$ (or k') ≤ 1.15

Table S5. Results of internal (train set (int) and cross validation (CV)) validation for binary classification models.

ML algorithm	Molecular feature	BA_{int}	MCC_{int}	ROC_AUC_{int}	BA_{CV}	MCC_{CV}	ROC_AUC_{CV}
RF	ECFP4	0.95	0.89	0.99	0.80 +/- 0.04	0.60 +/- 0.04	0.90 +/- 0.02
	MACCS2	0.93	0.84	0.98	0.79 +/- 0.03	0.60 +/- 0.03	0.88 +/- 0.02
	descriptors	1	1	1	0.81 +/- 0.02	0.63 +/- 0.02	0.90 +/- 0.02
	ECFP6	0.97	0.93	1	0.80 +/- 0.04	0.62 +/- 0.04	0.90 +/- 0.02
SVC	ECFP4	1	0.99	1	0.78 +/- 0.04	0.59 +/- 0.04	0.87 +/- 0.03
	MACCS2	0.96	0.91	0.98	0.77 +/- 0.02	0.56 +/- 0.02	0.84 +/- 0.02
	descriptors	1	1	1	0.80 +/- 0.03	0.61 +/- 0.03	0.87 +/- 0.02
	ECFP6	1	1	1	0.67 +/- 0.03	0.44 +/- 0.03	0.87 +/- 0.02
XGBoost	ECFP4	0.99	0.99	1	0.77 +/- 0.03	0.55 +/- 0.03	0.88 +/- 0.02
	MACCS2	0.93	0.84	0.98	0.77 +/- 0.02	0.56 +/- 0.02	0.87 +/- 0.01
	descriptors	1	1	1	0.80 +/- 0.02	0.61 +/- 0.02	0.89 +/- 0.01
	ECFP6	1	1	1	0.79 +/- 0.03	0.58 +/- 0.03	0.88 +/- 0.02
KNN	ECFP4	1	0.99	1	0.79 +/- 0.03	0.59 +/- 0.03	0.87 +/- 0.03
	MACCS2	0.96	0.92	1	0.77 +/- 0.02	0.55 +/- 0.02	0.84 +/- 0.01
	descriptors	1	1	1	0.64 +/- 0.03	0.34 +/- 0.03	0.82 +/- 0.03
	ECFP6	1	1	1	0.80 +/- 0.03	0.61 +/- 0.03	0.88 +/- 0.03
DNN	ECFP4	1	0.99	1	0.8 +/- 0.05	0.61 +/- 0.05	0.89 +/- 0.02
	MACCS2	0.96	0.9	1	0.81 +/- 0.03	0.63 +/- 0.03	0.9 +/- 0.01
	descriptors	1	1	1	0.84 +/- 0.01	0.69 +/- 0.01	0.91 +/- 0.01
	ECFP6	0.98	0.96	1	0.82 +/- 0.02	0.64 +/- 0.02	0.91 +/- 0.02

Table S6. Results of internal (train set (int) and cross validation (CV)) validation for multiclass classification models.

Model	ML algorithm	Molecular feature	BA_{int}	MCC_{int}	ROC_AUC_{int}	BA_{CV}	MCC_{CV}	ROC_AUC_{CV}
SIRT1/2	RF	ECFP4	0.93	0.89	0.99	0.74 +/- 0.02	0.63 +/- 0.02	0.9 +/- 0.01
		MACCS2	0.9	0.85	0.99	0.73 +/- 0.03	0.62 +/- 0.03	0.89 +/- 0.01
		descriptors	1	1	1	0.75 +/- 0.03	0.63 +/- 0.03	0.9 +/- 0.01
		ECFP6	0.94	0.91	1	0.75 +/- 0.02	0.64 +/- 0.02	0.9 +/- 0.01
	SVC	ECFP4	0.97	0.95	1	0.75 +/- 0.03	0.64 +/- 0.03	0.89 +/- 0.01
		MACCS2	0.97	0.94	0.99	0.72 +/- 0.03	0.6 +/- 0.03	0.86 +/- 0.01
		descriptors	1	0.99	1	0.73 +/- 0.05	0.61 +/- 0.05	0.88 +/- 0.02
		ECFP6	1	1	1	0.75 +/- 0.03	0.64 +/- 0.03	0.89 +/- 0.01
	XGBoost	ECFP4	0.99	0.99	1	0.75 +/- 0.02	0.64 +/- 0.02	0.9 +/- 0.01
		MACCS2	0.92	0.88	0.99	0.74 +/- 0.01	0.63 +/- 0.01	0.9 +/- 0.01
		descriptors	1	1	1	0.75 +/- 0.02	0.64 +/- 0.02	0.89 +/- 0.01
		ECFP6	0.9	0.84	0.98	0.74 +/- 0.01	0.63 +/- 0.01	0.9 +/- 0.01
	KNN	ECFP4	1	1	1	0.72 +/- 0.01	0.59 +/- 0.01	0.88 +/- 0.01
		MACCS2	0.97	0.95	1	0.69 +/- 0.03	0.56 +/- 0.03	0.86 +/- 0.02
		descriptors	0.85	0.76	0.96	0.67 +/- 0.03	0.54 +/- 0.03	0.86 +/- 0.01
		ECFP6	1	1	1	0.71 +/- 0.02	0.58 +/- 0.02	0.89 +/- 0.01
	DNN	ECFP4	1	1	1	0.75 +/- 0.03	0.63 +/- 0.02	0.9 +/- 0.02
		MACCS2	0.95	0.92	1	0.74 +/- 0.01	0.61 +/- 0.05	0.89 +/- 0.01
		descriptors	0.99	0.99	1	0.75 +/- 0.03	0.64 +/- 0.02	0.9 +/- 0.01
		ECFP6	0.98	0.99	1	0.75 +/- 0.03	0.63 +/- 0.01	0.9 +/- 0.01
SIRT2/3	RF	ECFP4	0.94	0.9	1	0.8 +/- 0.03	0.74 +/- 0.03	0.95 +/- 0.02
		MACCS2	0.94	0.89	0.99	0.78 +/- 0.01	0.72 +/- 0.01	0.93 +/- 0.02
		descriptors	1	1	1	0.8 +/- 0.03	0.74 +/- 0.03	0.94 +/- 0.02
		ECFP6	0.96	0.93	1	0.81 +/- 0.03	0.76 +/- 0.03	0.95 +/- 0.02
	SVC	ECFP4	1	1	1	0.71 +/- 0.03	0.65 +/- 0.03	0.94 +/- 0.03
		MACCS2	0.98	0.95	0.99	0.8 +/- 0.02	0.72 +/- 0.02	0.91 +/- 0.02
		descriptors	1	0.99	1	0.73 +/- 0.05	0.61 +/- 0.05	0.88 +/- 0.02
		ECFP6	1	1	1	0.78 +/- 0.03	0.7 +/- 0.03	0.94 +/- 0.02
	XGBoost	ECFP4	1	1	1	0.76 +/- 0.05	0.69 +/- 0.05	0.94 +/- 0.02

		MACCS2	0.98	0.95	1	0.78 +/- 0.04	0.7 +/- 0.04	0.92 +/- 0.02
		descriptors	1	1	1	0.79 +/- 0.01	0.72 +/- 0.01	0.94 +/- 0.01
		ECFP6	1	1	1	0.79 +/- 0.04	0.72 +/- 0.04	0.94 +/- 0.02
	KNN	ECFP4	1	1	1	0.77 +/- 0.05	0.7 +/- 0.05	0.92 +/- 0.03
		MACCS2	0.98	0.96	1	0.73 +/- 0.03	0.65 +/- 0.03	0.9 +/- 0.02
		descriptors	0.85	0.76	0.96	0.67 +/- 0.03	0.54 +/- 0.03	0.86 +/- 0.01
		ECFP6	1	1	1	0.77 +/- 0.04	0.69 +/- 0.04	0.92 +/- 0.03
	DNN	ECFP4	1	1	1	0.8 +/- 0.03	0.74 +/- 0.03	0.94 +/- 0.03
		MACCS2	1	1	1	0.78 +/- 0.03	0.74 +/- 0.03	0.94 +/- 0.02
		descriptors	1	1	1	0.8 +/- 0.03	0.76 +/- 0.03	0.95 +/- 0.02
		ECFP6	0.99	0.99	1	0.78 +/- 0.03	0.65 +/- 0.03	0.93 +/- 0.02

Table S7. External (test set) validation parameters of the multiclass SIRT1/2 models.

ML algorithm	Molecular feature	BA	MCC	ROC_AUC [a]	Precision [a]	Recall [a]	F1 [a]
RF	descriptors	0.85	0.77	0.96	0.84	0.85	0.84
	ECFP4	0.84	0.74	0.96	0.81	0.84	0.82
	MACCS2	0.82	0.72	0.95	0.81	0.82	0.81
	ECFP6	0.83	0.74	0.96	0.81	0.83	0.82
SVC	descriptors	0.86	0.79	0.96	0.85	0.86	0.86
	ECFP4	0.83	0.73	0.95	0.81	0.83	0.82
	MACCS2	0.82	0.72	0.94	0.8	0.82	0.81
	ECFP6	0.8	0.7	0.95	0.8	0.8	0.8
XGBoost	descriptors	0.84	0.76	0.95	0.83	0.84	0.84
	ECFP4	0.84	0.74	0.96	0.82	0.84	0.83
	MACCS2	0.83	0.73	0.95	0.81	0.83	0.82
	ECFP6	0.81	0.71	0.95	0.79	0.82	0.8
KNN	descriptors	0.8	0.69	0.93	0.78	0.8	0.79
	ECFP4	0.84	0.73	0.94	0.82	0.84	0.82
	MACCS2	0.84	0.75	0.94	0.82	0.84	0.83
	ECFP6	0.83	0.71	0.93	0.81	0.83	0.81
DNN	descriptors	0.84	0.77	0.93	0.84	0.84	0.84
	ECFP4	0.82	0.77	0.94	0.84	0.82	0.83
	MACCS2	0.82	0.73	0.92	0.81	0.82	0.81
	ECFP6	0.84	0.74	0.95	0.82	0.84	0.82

[a] Values are calculated using macro averages and one-vs-rest approach.

Table S8. The predictive performance parameters of the multiclass SIRT1/2 models on the decoys dataset.

ML algorithm	Molecular feature	BA	MCC	ROC_AUC [a]	Precision [a]	Recall [a]	F1 [a]
RF	descriptors	0.72	0.09	0.91	0.35	0.72	0.24
	ECFP4	0.82 (0.88)*	0.2 (0.73)*	0.92 (0.95)*	0.37 (0.72)*	0.82 (0.89)*	0.37 (0.79)*
	MACCS2	0.71	0.09	0.89	0.35	0.71	0.25
	ECFP6	0.8	0.16	0.91	0.36	0.8	0.34
SVC	descriptors	0.68	0.08	0.92	0.35	0.68	0.19
	ECFP4	0.7	0.09	0.9	0.35	0.7	0.23
	MACCS2	0.72	0.1	0.9	0.35	0.72	0.26
	ECFP6	0.69	0.08	0.91	0.35	0.69	0.22
XGBoost	descriptors	0.71	0.09	0.89	0.34	0.71	0.22
	ECFP4	0.75	0.12	0.93	0.35	0.75	0.28
	MACCS2	0.7	0.09	0.89	0.34	0.7	0.24
	ECFP6	0.73	0.11	0.91	0.35	0.73	0.28
KNN	descriptors	0.62	0.06	0.7	0.34	0.62	0.17
	ECFP4	0.69	0.08	0.81	0.34	0.69	0.22
	MACCS2	0.65	0.07	0.76	0.34	0.65	0.17
	ECFP6	0.67	0.08	0.81	0.34	0.67	0.21
DNN	descriptors	0.7	0.09	0.71	0.35	0.7	0.22
	ECFP4	0.67	0.08	0.87	0.34	0.67	0.22
	MACCS2	0.67	0.08	0.74	0.34	0.67	0.21
	ECFP6	0.68	0.08	0.87	0.34	0.68	0.21

[a] Values are calculated using macro averages and one-vs-rest approach.
* Values in brackets are obtained after applicability domain corrections.

Table S9. External (test set) validation parameters of the multiclass SIRT2/3 models.

ML algorithm	Molecular feature	BA	MCC	ROC_AUC [a]	Precision [a]	Recall [a]	F1 [a]
RF	descriptors	0.9	0.83	0.97	0.87	0.9	0.88
	ECFP4	0.83	0.73	0.96	0.8	0.83	0.81
	MACCS2	0.85	0.75	0.95	0.82	0.85	0.84
	ECFP6	0.83	0.74	0.95	0.8	0.83	0.81
SVC	descriptors	0.94	0.88	0.98	0.91	0.94	0.92
	ECFP4	0.77	0.71	0.97	0.83	0.78	0.8
	MACCS2	0.85	0.74	0.95	0.81	0.85	0.83
	ECFP6	0.82	0.74	0.95	0.82	0.82	0.82
XGBoost	descriptors	0.88	0.81	0.97	0.86	0.88	0.87
	ECFP4	0.84	0.73	0.96	0.81	0.85	0.82
	MACCS2	0.87	0.77	0.95	0.84	0.87	0.85
	ECFP6	0.8	0.71	0.96	0.79	0.81	0.8
KNN	descriptors	0.85	0.73	0.96	0.8	0.85	0.82
	ECFP4	0.85	0.74	0.93	0.8	0.85	0.82
	MACCS2	0.85	0.73	0.93	0.8	0.85	0.82
	ECFP6	0.85	0.73	0.93	0.79	0.85	0.82
DNN	descriptors	0.94	0.89	0.97	0.92	0.94	0.93
	ECFP4	0.84	0.75	0.95	0.81	0.84	0.83
	MACCS2	0.87	0.78	0.95	0.83	0.87	0.85
	ECFP6	0.88	0.81	0.97	0.85	0.88	0.86

[a] Values are calculated using macro averages and one-vs-rest approach.

Table S10. The predictive performance parameters of the multiclass SIRT2/3 models on the decoys dataset.

ML algorithm	Molecular feature	BA	MCC	ROC_AUC [a]	Precision [a]	Recall [a]	F1 [a]
RF	descriptors	0.66 (0.59)*	0.06 (0.12)*	0.93 (0.88)*	0.34 (0.47)*	0.66 (0.59)*	0.14 (0.34)*
	ECFP4	0.59	0.08	0.91	0.34	0.59	0.27
	MACCS2	0.65	0.06	0.92	0.34	0.65	0.17
	ECFP6	0.62	0.08	0.92	0.34	0.62	0.25
SVC	descriptors	0.62	0.06	0.95	0.34	0.62	0.02
	ECFP4	0.54	0.04	0.92	0.32	0.54	0.06
	MACCS2	0.58	0.05	0.93	0.34	0.58	0.08
	ECFP6	0.62	0.05	0.92	0.34	0.62	0.11
XGBoost	descriptors	0.7	0.07	0.92	0.34	0.7	0.19
	ECFP4	0.61	0.07	0.91	0.35	0.61	0.17
	MACCS2	0.69	0.07	0.92	0.34	0.69	0.21
	ECFP6	0.63	0.07	0.92	0.35	0.63	0.17
KNN	descriptors	0.64	0.05	0.71	0.34	0.64	0.15
	ECFP4	0.66	0.06	0.74	0.34	0.66	0.17
	MACCS2	0.61	0.04	0.66	0.34	0.61	0.12
	ECFP6	0.65	0.06	0.73	0.34	0.65	0.16
DNN	descriptors	0.72 (0.72)*	0.07 (0.1)*	0.87 (0.87)*	0.34 (0.34)*	0.72 (0.75)*	0.18 (0.2)*
	ECFP4	0.65	0.06	0.87	0.34	0.65	0.16
	MACCS2	0.67	0.06	0.87	0.34	0.67	0.18
	ECFP6	0.63	0.06	0.8	0.34	0.63	0.1

[a] Values are calculated using macro averages and one-vs-rest approach.
* Values in brackets are obtained after applicability domain corrections.

Table S11. Utility SIRT2i_Predictor for the processing of the previously published structure-based virtual screening results[20].

ID	SMILES	Activity (Inh% or IC50 (µM)) [a]	SIRT2 activity according to criteria of this work	Binary model predictions [b]	Predicted probability (binary model)[c]
NDJ134*	CCN1C(=O)c2cccc3c(S(=O)(=O)NCCc4ccccc4)ccc1c23	70.2%*	Twilight zone	Outside AD	0.455224
NDJ18	O=C(CCc1ccccc1)Nc1ccc(Cc2ccncc2)cc1	58.7 µM	Twilight zone	No	0.354493
NDJ85	Cc1ccccc1C(=O)NC(=S)NCc1ccc(-c2nn3c(C)nnc3s2)cc1	85.9 µM	Twilight zone	Outside AD	0.555415
NDJ11	Cc1ccccc1OCC(=O)Nc1ccc(Oc2ccc3c(c2)C(=O)NC3=O)cc1	55.6%	Twilight zone	No	0.383697
NDJ57	Cc1nnc2sc(-c3ccc(CNC(=O)c4ccc(-c5ccc(Cl)cc5)o4)cc3)nn12	47.2%	Twilight zone	Outside AD	0.415945
NDJ20	O=C(NCCc1ccccc1)c1ccc(NC(=O)c2cc(-n3cnnc3)ccc2Cl)cc1	45.1%	Twilight zone	No	0.335031
NDJ10	Cc1ccc(C)c(SCCc2nn3c(Cc4ccccc4F)nnc3s2)c1	40.5%	Twilight zone	Outside AD	0.549759
NDJ4	Cc1cc(=O)oc2cc(OC[C@H](O)Cn3c(C)nc4ccccc43)ccc12	31.8%	Inactive	Outside AD	0.518789
NDJ26	O=C1Nc2ccc(S(=O)(=O)N3CCN(c4ccccc4)CC3)c3cccc1c23	30.0%	Inactive	No	0.391046

[a] Inh% assays were performed at compounds concentration of 200 µM.
[b] No – not predicted to be active SIRT2 inhibitor; Yes – Predicted to be active SIRT2 inhibitor. For definition of "active" class see the Manuscript.
[c] The predicted probability for "active" class prediction could be between 0 and 1. If between 0.4 – 0.6, it is considered outside applicability domain (AD).
* Compound interfered with the fluorescent assay.

Table S12. Results of *in vitro* evaluation of the in-house compounds.

ID	SMILES	Activity (Inh% [a])	SIRT2 actiivty according to criteria of this work	Binary model predictions [b]	Predicted probability (binary model) [c]
316	COc1ccc([C@H]2Cc3cc(OC4O[C@H](CO)[C@@H](O)[C@H](O)[C@H]4O)cc(O)c3C(=O)O2)cc1	28.2%	Inactive	Outside AD	0.473878
1024*	/†	48.7% *	Twilight zone	No	0.294624
1558[3]	COc1ccc(Cn2cnc3c(Cl)nccc32)cc1	51.1%	Twilight zone	No	0.34941
1657[4]	COc1ccc(CN2NC([N+](=O)[O-])c3ccnc(NC4CCCCC4)c32)cc1	59.7%	Twilight zone	Outside AD	0.427429
Ex-527	NC(=O)C1CCCc2c1[nH]c1ccc(Cl)cc21	89.6%	Active	Yes	0.830218

[a] Inh% assays were performed at compounds concentration of 200 μM.
[b] No – not predicted to be active SIRT2 inhibitor; Yes – Predicted to be active SIRT2 inhibitor. For definition of "active" class see the Manuscript.
[c] The predicted probability for "active" class prediction could be between 0 and 1. If between 0.4 – 0.6, it is considered outside applicability domain (AD).
* Compound tested at 100 μM due to assay interference.
† The compound structure is available upon request since synthesis is yet to be published.

Supplementary Note S1 – Internal validation parameters

Following formula were used for calculation of internal validation parameters:

$$R_{int}^2 = 1 - \frac{\sum_{i=1}^{n_{int}}(y_i-\hat{y}_i)^2}{\sum_{i=1}^{n_{int}}(y_i-\bar{y})^2} \quad (1)$$

$$RMSE_{int} = \sqrt{\frac{\sum_{n=1}^{n_{int}}(y_i-\hat{y}_i)^2}{n_{int}}} \quad (2)$$

$$Q^2 = 1 - \frac{\sum_{i=1}^{n}(y_i-\hat{y}_{i/i})^2}{\sum_{i=1}^{n}(y_i-\bar{y})^2} \quad (3)$$

$$RMSE_{CV} = \sqrt{\frac{\sum_{n=1}^{n}(y_i-\hat{y}_{i/i})^2}{n}} \quad (4)$$

Where y_i represents experimental data values; $\hat{y}_i$ represents predicted data values; $\hat{y}_{i/i}$ response of the i-th object estimated by using a model obtained without using the i-th; n - number of objects.

For binary classification models, Matthews correlation coefficient (MCC) was calculated according to the equation (5):

$$MCC = \frac{(TP \times TN)-(FP \times FN)}{\sqrt{(TP+FP)\times(TP+FN)\times(TN+FP)\times(TN+FN)}} \quad (5)$$

Where *TP* stands for number of true positive predictions, *TN* stands for number of true negative predictions, *FP* stands for number of false positive predictions and *FN* stands for number of false negative predictions.

Supplementary Note S2 - Supplementary methods

***In vitro* enzymatic assays for SIRT2**. Deacetylation activities of SIRT2 in presence of in-house compounds were determined using previously described protocol for Fluor de Lys fluorometric assays (Enzo Life Sciences, Ann Arbor, MI, USA)[68]. Briefly, the reaction was started by incubating the enzyme (SIRT2) with the reaction mixture of acetylated peptide substrate BioMol KI179 for SIRT2 (0.7 Km: 198 µM for SIRT2), NAD^+ (0.9 Km: 547 µM for SIRT2), and 5% dimethyl sulfoxide (DMSO) or compounds in 5% final DMSO concentration. Incubation was done at 37 °C, for 1 hour. The developer and nicotinamide (2 mM in histone deacetylase (HDAC) assay buffer in total volume of 50 µl) were added and the incubation was continued for 45 min at 37 °C. The fluorescence was measured using EnVision 2104 Multilabel Reader (PerkinElmer, Waltham, MA, USA) with excitation and emission wavelengths of 370 nm and 460 nm, respectively. All

experiments were performed in triplicates. Compounds were screened on SIRT2 inhibitory activity at 200 μM to obtain percent of inhibition.
